# A Cross-Sectional Study to Ascertain the Effect of COVID-19 Pandemic on Regional Anaesthesia Practices Amongst Anaesthesiologists of India

**DOI:** 10.7759/cureus.28228

**Published:** 2022-08-21

**Authors:** Anju Gupta, Bhavya Krishna, Vishnu Narayanan, Shailendra Kumar, Nishkarsh Gupta, Amit K Malviya, Rajeev Kumar Malhotra, Lokesh Kashyap

**Affiliations:** 1 Anaesthesiology, Pain Medicine and Critical care, All India Institute of Medical Sciences, New Delhi, New Delhi, IND; 2 Anaesthesiology, Vardhman Mahavir Medical College and Safdarjung Hospital, Delhi, IND; 3 Anaesthesiology, Pain Medicine and Critical Care, AII India institute of Medical Sciences, New Delhi, New Delhi, IND; 4 Anaesthesiology, All India Institute of Medical Sciences, New Delhi, New Delhi, IND; 5 Onco-Anaesthesiology and Palliative Medicine, All India Institute of Medical Sciences, New Delhi, New Delhi, IND; 6 Anaesthesiology and Critical Care, All India Institute of Medical Sciences, New Delhi, New Delhi, IND; 7 Statistics, All India Institute of Medical Sciences, New Delhi, New Delhi, IND; 8 Anaesthesiology, Pain Medicine and Critical Care, All India Institute of Medical Sciences, New Delhi, New Delhi, IND

**Keywords:** pandemic, covid-19 retro, personal protective equipment, electronic mail, covid 19, anesthesiologists, aerosols

## Abstract

Background and aims

Regional anaesthesia has been advocated as a preferred modality during the coronavirus disease 2019 (COVID‑19) pandemic, but pursuing regional anaesthesia during COVID-19 is challenging. Our cross-sectional survey aimed to analyze the challenges in conducting regional anaesthesia and the alterations in practices imposed by the COVID‑19 pandemic across the nation.

Material and methods

The questionnaire was validated by seven experts. Following ethical approval and trial registration, this Google Forms-based survey was circulated to anaesthesiologists across the country via emails over 3 months (April 2021 to June 2021). Weekly reminders were sent to the non-responders till the desired sample size was attained, after which the survey was closed and responses were analyzed.

Results

Five hundred and thirty-two of 1100 anaesthesiologists completed the survey (48.3% response rate). Among the 532 respondents, 65.8% reported an increase in the use of regional anaesthesia due to the pandemic, with 77.4% reporting a change in practice. Almost 90% of the respondents used a dedicated operation theatre for all infected patients. Most respondents (75%) used disposable plastic drapes (75%) and full personal protective equipment (PPE) for COVID-19-positive patients during the procedure. However, using PPE resulted in poor vision due to fogging and multiple attempts and increased performance duration. Most respondents (74.4%) used gloves to maintain ultrasound probe sterility, while many other respondents (65.7%) used a sterile camera cover for the same. Many respondents ordered inflammatory markers during preoperative evaluation.

Conclusion

The present survey revealed that there was increased utilization of regional anaesthesia with increased utilization of PPE, sterility routines, and ordering of investigations. The use of PPE led to many challenges while performing regional anaesthesia. However, many deviations were identified from the accepted recommendations, and one needs to be aware of proper practices to achieve optimal patient outcomes and provider safety.

## Introduction

The coronavirus disease 2019 (COVID-19) tested both our vulnerability and resilience in all aspects of life, including anaesthesia practice and training. Anaesthesiologists across the world are still struggling to adapt to the COVID-19 pandemic in every possible manner to optimize patient and provider safety. Anaesthesia techniques that decrease healthcare workers’ interactions with potentially infectious patients are favoured [1]. Social distancing and rational use of personal protection equipment (PPE) recommended by the World Health Organization has been universally followed. Special attention was given to aerosol-generating procedures (AGPs) such as airway management, positive pressure ventilation, extubation, etc., for surgical procedures [2]. AGPs pose a greater hazard for healthcare workers to contracting an infection. This is of particular concern in the field of anaesthesia because of the vast number of AGPs performed by them. Considering the possibility of facing asymptomatic infections, precautions had to be taken in the management of all the patients. A lack of appropriate protection may lead to healthcare workers getting infected, and infected symptomatic personnel signifies a decrease in the hospital workforce.

Regional anaesthesia (RA) has well-known benefits over general anaesthesia even during the non-COVID-19 era and is known to improve many patient-related outcomes such as superior analgesia, reduced postoperative complications, fast-track patient recovery, decreased hospital length of stay and possible favourable benefits on patient immunity, but the COVID-19 pandemic has seen unprecedented favouring of RA [3]. Throughout the pandemic, we rediscovered the important role of RA as a modality to decrease COVID-19 spread during AGPs. The European and American Societies of Regional Anesthesia recommend performing RA as the sole anaesthetic technique for managing COVID-19-positive/suspected patients undergoing surgery to improve healthcare safety for both patients and providers [4].

However, COVID-19 has posed many challenges in pursuing RA. The use of PPE can diminish visibility, mobility, and communication, thereby increasing technical difficulties. The reduced footfall has decreased the number of procedures and impacted RA-related teaching, training, and research. Coagulation abnormalities in many COVID-19 patients may impact the choice of central neuraxial anaesthesia. The concerns of contamination of the ultrasound machine and other RA equipment have also been expressed. No survey has dealt with these issues to date.

Hence, the present survey was devised keeping in mind the challenges being faced by anaesthesiologists during RA practice and the impact of the pandemic on its choice, practice, training among the anaesthesiologists, as well as the strategies necessary to counter the hindrances and improve the safe utilization of RA during the pandemic time.

## Materials and methods

This cross-sectional descriptive study based on a Google Form was undertaken after due approval from the Institutional Ethics Committee of a tertiary care teaching hospital and the survey was circulated after registering with the national clinical trials registry (REF/2020/05/033338). The participation of medical professionals in this study was voluntary. The form was circulated through email to be filled out by participants all over the country who were anesthesiologists with any experience working in government, private, autonomous institutions, or as a freelancer in India and willing to participate in the study. A semi-structured questionnaire consisting of qualitative and quantitative questions was developed by using Google Forms to assess the challenges faced by them in pursuing RA during the COVID era and any related change in the utilization, use of PPE, and its associated difficulties, changes in techniques used, and training/research activities related to RA due to the insufficiencies which had arisen during the pandemic and the strategies that were being adopted to overcome those challenges (Appendices). A snowball sampling technique was used, and the participants were encouraged to roll out the survey to as many people further as possible. On receiving and clicking the link, the participants got auto-directed to a brief mention of the need for the survey. In addition, participant consent was included in the form immediately after this information. After they accepted to take the survey, a set of several questions appeared sequentially. The survey did not contain any questions that could disclose the subject’s or the institute’s identity. Non-responders received a maximum of three e-mails as two weekly reminders. Once the desired sample size was attained, the survey was closed for further responses. The results of the survey were kept confidential. 

Validity and reliability of the study 

Seven out of 10 experts across specialities responded to our request for assessment and grading as per the instructions provided to them. The mean of the item-wise content validity index (I-CVI) for relevance, simplicity, clarity, and ambiguity was 0.98, 0.99, 0.90, and 0.94, respectively. In our study, the Kappa statistic of each question in relevance, simplicity, clarity, and ambiguity was between 0.75-1, which is excellent as per the strength of agreement classified by Cicchetti [5]. According to Lynn, an I-CVI of >0.78 and a scale-CVI (S-CVI)/average of 0.9 are acceptable when more than six experts graded the tool [6]. This condition was also fully satisfied during the validation process.   

 Statistical analysis

The data were entered in a Microsoft Excel chart (Microsoft Corporation, Redmond, USA) and the results were interpreted accordingly. Percentages and frequencies were used in the conclusion of the data. The sample size was calculated to be 384, considering 16,000 anaesthesiologists and assuming that regional anaesthesia practice has been affected in the case of at least 50% of the anaesthesiologists, with a margin of error of 5% and confidence interval of 95%. We planned to recruit 512 participants considering a 25% non-response rate. The associations were calculated using the Chi-square test. The data was entered in a Microsoft Excel chart and the results were analyzed by using Statistical Package for Social Sciences (SPSS) version 22 (IBM Corp., Armonk, USA).

## Results

Responses were collected from 534 out of 1100 eligible respondents who were contacted (48.3% response rate), and after excluding two incomplete responses, 532 were evaluated. As such, 86% of the respondents were from medical colleges and hospitals, 7.9% from corporate hospitals, and the rest were freelance anaesthesiologists or from non-teaching government hospitals (Table 1).

**Table 1:** Demographics and general profile of the participants *RA: regional anesthesia

Variable	Categories	Percentage (number)
Type of institution	Medical college and hospital	86%, (458)
	Government non-teaching hospital	2%, (11)
	Corporate hospital	7.89%, (42)
	Freelancing practitioner	3.9%, (21)
Years of experience	<5 years	68% (362)
	5-10 years	19.9% (106)
	10-15 years	5.6% (30)
	>15 years	6.4% (34)
Percentage of surgeries under RA*	<20%	27.6%, (147)
	20-40%	42.7%, (227)
	40-60%	20.7%, (110)
	>60%	9%, (48)
Patients undergoing surgery that receive RA for postoperative analgesia	<10%	25%, (133)
	10-20%	41%,(218)
	20-30%	13.9%, (74)
	>30%	20.1%, (107)

Among the 532 respondents, 65.8% reported an increase in the use of RA due to the COVID-19 pandemic, with 77.4% reporting a change in practice in performing RA, such as the use of PPE, asepsis, etc. (Table 2).

**Table 2:** Changes in the practice of RA during the pandemic RA: regional anaesthesia; PPE: personal protective equipment; GA: general anaesthesia; TLC: total leucocyte count; Hb: haemoglobin; CXR: chest X-ray; KFT: kidney function test; LFT: liver function test; FFP: filtering facepiece; OR: operating room; PACU: post-anaesthesia care unit; LA: local anaesthetics;  SpO2: arterial oxygen saturation; 2D-ECHO: echocardiograph; ECG: electrocardiogram

Variable	Categories	Percentage (number)
Change in the incidence of using RA techniques for surgery during the pandemic	Decreased	13.5%, (72)
	Increased	65.8% (350)
	No change	20.7%, (110)
Change in an overall practice of RA with regards to the utilization, use of PPE, asepsis, disinfection, monitoring, type of technique used for postoperative analgesia in your institute	Yes	77.4%, (412)
	No	22.6%, (120)
Mode of anaesthetic technique preferred in COVID-19 patients undergoing surgery	General Anesthesia	7%, (37)
	Regional Anesthesia	77.6% , (413)
	No preference for any technique over the other	15.4%, (82)
Difference in asking for COVID-19 testing in cases scheduled under RA	No difference	62.4%, (332)
	More stringent for GA cases	37.6%, (200)
Availability of designated Operation theatre to perform surgeries of COVID confirmed/suspected patients	No	10% , (53)
	Yes, but with no negative pressure system	54.7%, (291)
	Yes, with negative pressure system	35.4%, (188)
Anesthesia practice		
Preoperative investigations routinely done in all adult COVID-19 patients posted for surgery under RA	TLC with Hb	100%, (532)
	Coagulation profile	94.3%, (502)
	Platelet count	89.8%, (478)
	Inflammatory markers	21.4%, (114)
	CXR	89.8%, (478)
	CT Chest	36.8%, (196)
	ECG	61.4%, (327)
	KFT	45.3%, (241)
	LFT	37.2%, (198)
	Temperature	59.3%, (316)
	SpO2 (Oxygen saturation)	47.9%, (255)
	2D Echocardiography	8.6%, (46)
What is your practice of sedation for all COVID 19 patients undergoing surgery under RA?	Avoided unless required	63%, (335)
	Yes in all cases	37%, (197)
What type of mask is applied to COVID 19 patients undergoing surgery under RA?	No mask applied	3.6%, (19)
	Yes, surgical mask	54.5%, (290)
	Yes, surgical 3 layered mask	24.6%, (131)
	Yes, N95/FFP 3 mask	17.3%, (92)
Do you have intralipid stored inside the COVID designated OR in your hospital?	Yes	69.9%, (372)
	No	30.1%, (160)
Where do you monitor COVID-positive patients during the immediate postoperative period?	COVID-designated ward	45.3%, (241)
	PACU	6.8%, (36)
	Within the COVID designated OR	47.9%, (255)
Training methods provided for residents for performing RA in your hospital during the peak of the COVID pandemic	Web-based learning	61%, (325)
	Deliberate practice on pts under the supervision of faculty	27.2%, (145)
	Simulation-based learning	17.2%, (92)
	Cadaver/Phantom	3.5%, (19)
	Workshops	12.6%, (67)
	No training	3%, (16)
Issues with PPE		
Problems faced while performing a Central neuraxial with PPE	Poor vision due to fogging	91.3%, (486)
	Multiple attempts	62.5%, (270)
	increased duration of performance	56%, (298)
	Difficulty in palpation	26.12%, (139)
	increased incidence of dural puncture while putting epidural	5.6%, (30)
	Failure to block	5%, (27)
What issues have you faced in performing peripheral nerve blocks with PPE?	Poor visibility due to fogging	81.9%, (436)
	Multiple attempts	49.6%, (264)
	increased performance time	46.9%, (250)
	Poor ultrasound images	14%, (75)
	Increased failure rates	14.34%, (71)
	Increased vol of LA	3.3%, (18)
	Wrong side block	1.8%, (10)
	Nerve Injury	1.6%, (9)
	Haematoma	0.9%, (5)

A bulk of the anaesthesiologists, 77.6%, preferred RA for anaesthetizing patients with suspected/confirmed COVID-19 infection. The majority of the respondents (90.1%) had a separate dedicated operation theatre (OT) for covid patients.

Only 3.6% of respondents did not apply a protective mask (surgical mask/N95) over suspected/confirmed COVID-19 patients coming for surgery under RA. Table 3 shows all the changes and practices during the performance of central neuraxial blockade (CNB) and peripheral nerve blocks during the covid 19 pandemic.

**Table 3:** Change in the practice of central neuraxial block and peripheral nerve block in COVID-19 patients during the pandemic CNB: Central neuraxial block; PNB: peripheral nerve block; PCA: patient-controlled analgesia

Variable	Categories	Percentage (number)
Disinfection of the skin prior to performing CNB in COVID-19 patients	Povidone iodine	37.6%, (200)
	Chlorhexidine with isopropyl alcohol	21.8%, (116)
	Sterillium	17.9%, (96)
	Both povidone and chlorhexidine	22.7%, (120)
Position do you perform CNB in a COVID-19 patient	Lateral	8.8%, (47)
	Sitting	52.6%, (280)
	Depends on patient	38.6%, (205)
Size of needle used to perform spinal anaesthesia	23G	5.4%, (29)
	25G	71.2%, (379)
	26G	19.4%, (103)
	27G	4%, (21)
Type of needle do you use for spinal anaesthesia in a COVID-19 patient	Pencil	9.4%, (50)
	Quincke	88.9%, (473)
	Either	1.7%, (9)
Adjuvants used in spinal anaesthesia for COVID-19 patients	Fentanyl	89%, (474)
	Morphine	42.3%, (225)
	Clonidine	15%, (80)
	Dexmedetomidine	7.5%, (40)
	Buprenorphine	1.6%, (9)
	Butorphanol	0.56%, (3)
	Nalbuphine	0.18%, (1)
	Combination	0.9%, (5)
	No adjuvants	3.38%, (18)
Prophylactic measures do you use to prevent hypotension following spinal anaesthesia in a COVID-19 patient	Preloading	34.8%, (185)
	Co-loading	45.9%, (244)
	Pre or co-loading	7.4%, (40)
	Prophylactic vasopressors	5.1%, (27)
	No active interventions	6.8%, (36)
Needle size is used to perform epidural anaesthesia in COVID-19 patients	16G	22.2%, (118)
	18G	62.2%, (331)
	Either	15.6%, (83)
Method used for epidural space identification in COVID-19 Patients	Loss of resistance to air	88.7%,(472)
	Loss of resistance to saline	5.6%, (30)
	Loss of resistance to air/saline	1.5%, (8)
	Hanging drop	2.9%, (15)
	Ultrasound	1%, (5)
	Others	0.4%, (2)
Management of postdural puncture headache	Conservative management - Analgesics, Caffeine, fluids, rest, lying position	28.7%, (153)
	Dexamethasone	0.18%, (1)
	Bilateral Greater Occipital block	29.1%, (155)
	Sphenopalatine block	11%, (59)
	Epidural blood patch	25%, (133)
	Occipital block + blood patch	2.25%, (12)
	Sphenopalatine + blood patch	1.3%, (7)
	Occipital block + sphenopalatine block	0.75%, (4)
	Occipital + sphenopalatine + blood patch	0.37%, (2)
	Not encountered	1.1%, (6)
Mode of drug delivery through epidural for postoperative pain relief	Repeated bolus at regular intervals	2.9%, (16)
	Continuous infusion	16.4%, (87)
	Bolus when patient complaints	28.4%, (151)
	PCA	2.8%, (15)
	Yes, as continuous infusion, Bolus when patient’s complaints	1%, (5)
	continuous infusion with bolus at regular intervals	48.5%, (258)
Which investigations do you check prior to removal of epidural catheter	Coagulation profile	48.5%, (258)
	Platelet count	19.3%, (103)
	Both	27.8%, (148)
	Others	4.3%, (23)
Technique commonly used to perform PNB	Combined USG with nerve stimulator	26.5%, (141)
	Landmark	16.4%, (87)
	Nerve stimulator	10.2%, (54)
	USG guided	47%, (250)
Adjuvant commonly used for performing PNB	Opioids	55%, (293)
	Clonidine	20.6%, (110)
	Dexmedetomidine	9.2%, (49)
	Dexamethasone	30.6%, (163)
	Adrenaline	0.18%, (1)
	Sodium bicarbonate	0.18%, (1)
	Tramadol	0.36%, (2)
	None	1.8%, (10)
Method used for disinfection of the skin prior to performing PNB	Chlorhexidine with isopropyl alcohol	38.2%, (203)
	Pure alcohol-based solution	15%, (80)
	Povidone iodine with alcohol-based solutions	0.4%, (2)
	Povidone iodine	46.4%, (247)
Do you insert of perineural catheters for postoperative analgesia	Yes	20.3%, (108)
	No	79.7%, (424)
Duration for keeping perineural catheters	<24 hours	50.6%, (269)
	24-72 hours	2.1%, (11)
	>72 hours	47.3%, (252)
Blocks preferred for upper limb surgery	Supraclavicular	86%, (458)
	Interscalene	17.6%, (94)
	Infraclavicular	7.3%, (39)
	Axillary	16.7%, (89)
	Individual nerve blocks	2.4%, (13)
Who performs the block in COVID-19 positive patients	Consultant	13.7%, (73)
	Consultant, Senior Resident	3.9%, (21)
	Senior Resident	63.7%, (339)
	Post graduate Trainee	13.5%, (72)
	Senior Resident, Post graduate Trainee	3.6%, (19)
	Any of the above	1.5%, (8)

The majority of respondents (75%) used disposable plastic for COVID-19-positive patients for performing RA.

Poor vision due to fogging, multiple attempts, and increased duration of performance were the most common issues faced due to the use of PPE while performing regional anaesthesia. The majority of respondents (74.4%; 396) used gloves as the measure to maintain ultrasound sterility in the COVID-designated OT, while a large number of participants (65.7%) used a sterile camera cover for the same. (Table 4).

**Table 4:** Sterility, cleaning, and disposal RA: regional anaesthesia; USG: ultrasonography

Variable	Categories	Percentage (number)
Kind of drapes are used while performing RA	Disposable plastic drapes	75%, (399)
	Disposable paper drapes	11.8%, (63)
	Reusable woollen drapes	8.8%, (47)
	Antimicrobial impregnated drapes	2.6%, (14)
	Any of the above	1.8%, (9)
Disposal of sharps and needles	Collected in separate tamper-proof bags, autoclaved and recycled	13.7%, (73)
	Collected in separate tamper-proof bags, autoclaved and shredded	40.6%, (216)
	Not sure	28.6%, (152)
	Incineration	16.5%, (88)
	Don’t know	0.6%, (3)
Methods used for cleaning the operating room after the surgery of a COVID-positive patient	Fogging with Hydrogen peroxide vapour followed by segregation	39.8%, (212)
	wiping floors and solid surfaces with sodium hypochlorite solution	37.6%, (200)
	wiping floors and solid surfaces with 75% alcohol	46%, (245)
	Disinfection of metallic equipment with sodium hypochlorite solution	55.26%, (294)
	Fumigation with formaldehyde	0.3%, (2)
	Fogging with Viricidal solution	0.18%, (1)
	25-30 air exchange	0.18%, (1)
Measures to maintain ultrasound sterility	Sterile gloves	74.4%, (396)
	sterile jelly	62%, (330)
	Camera Cover	65.78%, (350)
	Dipping into povidone iodine	34%, (181)
	Chlorhexidine rubs	16.9% (90)
	Antibiotic impregnated plastic cover	10.15%, (54)
	no USG	3.9%, (21)
	No block in COVID-positive patient	0.18%, (1)

The association of demographic variables with changes in practice during the COIVD-19 pandemic and other variables has been summarized in Table 5. 

**Table 5:** Association of demographic variables, utilization of RA for surgery and analgesia with the changes in practice during the COVID-19 pandemic GA: general anaesthesia; RA; regional anaesthesia; ASA: American Society of Anesthesiologists; OT: operation theatre *P-value: Chi-Square test

Questions	Possible categories	Number (percentage)													
			Type of Institute			Year of experience			% of Surgery Under RA				% of Surgery RA for Analgesia		
		Total (n=532)	Academic (n=459)	Non-academic (n-73)	P-value*	<5 years (n=362)	>=5 years (n-170)	P-value*	<20% (n=147)	20-40% (n=227)	>=40% (n=158)	P-value*	<20% (n=133)	>=20% (n=399)	p-value*
Change in the incidence of using RA for surgery	Decreased	72(13.5)	67(14.6)	6(8.2)	0.091	60(16.6)	13(7.6)	0.004	9(6.1)	35(15.4)	29(18.4)	0.067	12(9.0)	61(15.3)	<0.001
	Increased	350(65.8)	304(66.2)	46(63.0)		238(65.7)	112(65.9)		111(75.5)	156(68.7)	83(52.4)		98(73.7)	252(63.2)	
	No change	110(20.7)	88(19.2)	21(28.8)		64(17.7)	45(26.5)		27(18.4)	36(15.9)	46(29.1)		23(17.3)	86(21.6)	
Change in an overall practice of techniques for postoperative analgesia	Yes	412(77.4)	356(77.6)	56(76.7)	0.872	275(76.0)	137(80.6)	0.234	29(19.7)	57(25.1)	34(21.5)	0.811	31(23.3)	89(22.3)	0.445
	No	120(22.6)	103(22.4)	17(23.3)		87(24.0)	33(19.4)		118(80.3)	170(74.9)	124(78.5)		102(76.7)	310(77.7)	
Mode of anaesthetic technique preferred in COVID 19 patients undergoing surgery	GA	37(7.0)	30(6.5)	7(9.6)	0.594	24(6.6)	13(7.6)	0.882	15(10.2)	12(5.3)	10(6.3)	0.014	10(7.5)	27(6.8)	0.001
	RA	413(77.6)	359(78.2)	54(74.0)		283(78.2)	130(76.5)		119(81.0)	185(81.5)	109(69.0)		113(85.0)	300(75.2)	
	No preference	82(15.4)	70(15.3)	12(6.4)		55(15.2)	27(15.9)		13(8.8)	30(13.2)	39(24.7)		10(7.5)	72(18.0)	
Any difference in COVID-19 testing in cases scheduled under RA vs GA	No difference	332(62.4)	280(61.0)	52(71.2)	0.094	233(64.4)	71(41.8)	0.174	67(45.6)	147(64.8)	118(74.7)	<0.001	55(41.4)	277(69.4)	<0.001
	More stringent for GA cases	200(37.6)	179(39.0)	21(28.8)		129(35.6)	99(58.2)		80(54.4)	80(35.2)	40(25.3)		78(58.6)	122(30.6)	
Availability of designated OT for COVID patients	No	53(10.0))	26(5.7)	27(37.0)	<0.001	17(4.7)	36(21.2)	<0.001	15(10.2)	7(3.1)	31(19.6)	<0.001	25(18.8)	28(7.0)	<0.001
	Yes, but with no negative Pressure system	291(54.7)	162(35.3)	26(35.6)		129(35.6)	59(34.7)		22(15.0)	80(35.2)	86(54.4)		25(18.8)	163(40.9)	
	Yes , with negative Pressure system	188(35.4)	271(59.0)	20(27.4)		216(59.7)	75(44.1)		110(74.8)	140(61.7)	41(25.9)		83(62.4)	208(52.1)	
Preoperative investigations routinely done in all COVID-19 confirmed/suspected young adult (18-60) ASA-I patients posted for surgery under RA	Avoided unless required	335(63.0)	279(60.8)	56(76.7)	0.009	217(59.9)	118(69.4)	0.035	55(37.4)	137(60.4)	143(90.5)	<0.001	63(47.4)	272(68.2)	<0.001
	Yes in all cases	197(37.0)	180(39.2)	17(23.3)		145(40.1)	52(30.6)		92(62.6)	90(39.6)	15(9.5)		70(52.6)	127(31.8)	
Epidural analgesia for postoperative pain relief in COVID-positive patients	Infusion	352(66.2)	297(64.7)	55(75.3)	0.074	222(61.3)	130(76.5)	0.001	113(76.9)	130(57.3)	109(69.0)	<0.001	100(75.2)	252(63.2)	0.011
	Bolus	180(33.8)	162(35.3)	18(24.7)		140(38.7)	40(23.5)		34(23.1)	97(42.7)	49(31.0)		33(24.8)	147(36.8)	

## Discussion

The results of the present survey revealed that there was increased utilization of RA and the pandemic necessitated a change in the practice of RA. Most participants reported a preference for RA for a known COVID-19 patient and had a separate, dedicated OT for covid patients. Increased challenges in performing RA were reported due to the use of PPE and many RA practices were changed or adapted to the needs of the situation. 

Aerosol-generating procedures (AGPs), such as intubation, non-invasive positive pressure ventilation, cardiopulmonary resuscitation, and bronchoscopy, pose a greater risk for healthcare workers contracting an infection [7]. This is of particular concern in the field of anaesthesia because of the vast number of AGPs routinely performed by them during general anaesthesia (GA). The use of RA mostly precludes the use of AGPs to a large extent and has gained huge favour during the COVID-19 pandemic. In COVID-19 infected/suspected patients during the pandemic, the role of RA has been emphasized over GA whenever possible [3]. Several guidelines for RA practice in the COVID-19 pandemic are available from various societies like the Indian Society of Anesthesiologists (ISA) [8], Association of Regional Anesthesia (AORA) [9] and American Society of Regional Anesthesia-European Society of Regional Anesthesia (ASRA-ESRA) [4] guidelines reiterate the same. This is supported in our survey where we found the majority of the respondents preferred RA over GA during the COVID-19 pandemic. All the above guidelines/recommendations/advisories also encourage modifications and add-ons to the present RA practices, and 77.4% of our respondents reported a preference along with a change in the RA practice, avoiding GA and sedation whenever possible.

During peaks/community transmission stages of the pandemic, patients should undergo COVID-19 testing irrespective of the type of anaesthesia planned. Our survey showed that testing is being done in all patients but 38% of anaesthesiologists have responded that the testing is done more stringently in patients who are planned for GA as compared to RA. This might be because of the assumption that no AGP occurs while a surgery is being done under RA and RA leads to lesser disruption of the patient's systemic physiology and lesser complications compared to GA.

Almost 90% of the respondents used a dedicated OT for all confirmed or suspected COVID-19 infected patients. ISA advisory encourages the same along with recommending that these OTs should be well labelled [8]. It is recommended that these dedicated OTs should be negatively pressurized to reduce aerosol spread. In the Indian setting, the lack of a negatively pressurized OT system is not uncommon and, in such settings, it is advisable to have minimal staff inside the OT and to switch to the positive pressure systems and air conditioning before transferring the patient.

As per our survey, total leukocyte count with differential count, platelet count, coagulation profile, and chest X-ray have been asked by most of the anaesthesiologists in COVID-19-positive/suspected patients along with oxygen saturation and temperature monitoring. Inflammatory markers were done by 22% of the anaesthesiologists. The wide variability in the investigations done preoperatively may be because of the different institutional protocols followed locally. Low platelet count is associated with an increased risk of severe disease and mortality in patients with COVID-19 [10]. On the other hand, thrombotic complications are also seen in patients with COVID-19 and 20% of them have severe coagulation abnormalities and disorders with the risk of developing disseminated intravascular coagulation [11]. Thus, the perioperative investigation should be individualized and the use of regional, especially central neuraxial blockade, should be undertaken with caution.

Oxygen therapy via non-invasive ventilation (NIV) and high-flow nasal cannula increases the risk of aerosolization and must also be avoided when providing RA to COVID-19 patients whenever possible as it is considered an AGP. An oxygen mask with the minimum flow to avoid hypoxia is desirable during surgery under RA as increased flow increases the dispersal distances of the exhaled contagious air suggested by the ESRA-ASRA joint statement [4]. However, only 15% of the anesthesiologists who participated in our survey avoided oxygen supplementation and 33% of them used facemasks with higher flows. This practice is unfavourable not only because it increases the transmission risk, but also because it increases oxygen usage. It is recommended that all patients wear a surgical mask/N95 mask to avoid aerosol spread [8]. We found that 75% of the anaesthesiologists preferred surgical masks for their patients and 17% applied N-95/FFP-3 masks on their patients.

About 84% of the respondents donned level-3 PPE before performing RA. This might be because the majority of the responders work in medical colleges and probably would have easier access to PPE compared to others. Level-3 PPE appears to reduce the risk of transmission to anaesthetists who are exposed to mildly symptomatic surgical patients [12]. N-95 masks were preferred by 57% of the anaesthesiologists. Eye protection with face shields was preferred by 62.4%, while eye goggles were preferred by 39% of the anesthesiologists. Wearing PPE while performing a procedure can have a negative impact due to factors like fogging, decreased visual and auditory acuity, mobility, and communication. The common problems included poor vision due to fogging in goggles/face shields(91%), increased duration (56%), repeated attempts of needling (50%), difficulty in palpating intervertebral space during neuraxial blocks (26%), increased incidence of dural puncture with epidural (5%) and block failure (5%). Other issues faced while performing blocks with PPE included poor ultrasound images, IV injection of local anaesthetics, haematoma, increased volume requirement of local anaesthetics, wrong-sided blocks and nerve injury, more error making, etc. [13].

Type, size, and position while performing neuraxial block does not have clinical significance during the COVID-19 pandemic, although dripping of cerebrospinal fluid (CSF) after lumbar puncture should be avoided as the virus has been isolated from the CSF of patients with COVID-19 encephalitis. AORA and ESRA-ASRA [5] also recommend that CSF contamination should be avoided [14].

Adequate dosage and careful selection of local anaesthetic and adjuvant should be done to avoid conversion to GA and facilitate effective block duration. ASRA-ESRA [4] does not recommend any dose adjustment in spinal anaesthesia. They also do not recommend the use of adjuvant opioids. However, in our survey, 89% of anaesthesiologists prefer using fentanyl in spinal anaesthesia, and 42% use morphine. Intrathecal opioids, especially morphine, should be used cautiously in these patients to avoid respiratory depression and the need for airway manipulation and they should be monitored postoperatively for delayed respiratory depression. Though there are no studies regarding the incidence of respiratory depression following intrathecal opioids in COVID-positive patients, there can be increased risk theoretically in these groups of patients due to their associated respiratory illness. Non-opioid adjuncts should be preferred if needed [15].

For postoperative pain relief with epidural analgesia, the majority of the anaesthesiologists in the survey preferred bolus doses, with 49% preferring repeat boluses at the regular interval while 28.5% prefer bolus when the patient complains of pain; 18% preferred continuous infusion, and the rest, patient-controlled analgesia (PCA). However, ASRA-ESRA joint statement recommends the use of continuous infusion over bolus as it decreases frequent patient contact [4]. This may not be possible in every setup because of the lack of personnel and resources for continuous monitoring.

There is no consensus on the investigations required before the removal of epidural catheters in COVID-positive patients. However, it is advisable to get platelet count and coagulation profile in these patients because of the risk of thrombocytopenia, use of anticoagulants, and coagulopathy. ASRA guidelines for anticoagulation and RA should be followed [16]. In our survey, 48% of the anaesthesiologists preferred doing a platelet count and 77% preferred doing a coagulation profile before epidural catheter removal, although other investigations such as thromboelastogram (TEG) usage have also been reported [17].

Zhang et al reported an increased incidence of hypotension with the central neuraxial blockade in parturient COVID-positive patients [18]. Co-loading followed by preloading were the most used methods to prevent hypotension following spinal anaesthesia by the respondents and only 7% used prophylactic vasopressors though no recommendation regarding the management of CNB-related hypotension exists at present.

When asked regarding the measures that are likely to be taken to manage post-dural puncture headache (PDPH) if conservative management fails, 32.5% of anaesthesiologists preferred bilateral greater occipital nerve block (GONB), 29% preferred epidural blood patch, and 13.5% preferred sphenopalatine block. The rest 28% preferred continuing conservative management. ASRA-ESRA joint statements [4] recommend avoiding sphenopalatine ganglion block (SGB)** **as it is a potential AGP. They also raise the concern of injecting viremic blood in epidural space can have potential consequences. So, it has been advised to defer blood patches until the patient recovers from infection unless the headache is severe [19]. GONB is becoming increasingly popular to manage PDPH in COVID-19 patients as also evidenced in our survey [20].

Ultrasound-guided blocks increase the success rate of the block by accurate needle placement. The accuracy is increased by the use of a concomitant nerve stimulator. It also decreases the volume of local anaesthetic required as compared to nerve stimulator alone /landmark guided methods. This is of particular importance in COVID-positive patients as block failure and conversion to GA are least favoured [21]. In our survey, 47% of anaesthesiologists preferred ultrasound-guided blocks, and 26.6% preferred combining ultrasound with nerve stimulators. With growing surgical wait times, concerns related to AGPs, and recommendations to avoid GA when feasible, USG-guided regional blocks have been favoured as a principal anaesthetic modality for certain surgeries [22]. 

The use of a long-acting local anaesthetic prolongs the anaesthetic effect of RA. Besides, a safe and sufficient dose of local anaesthetic should be used [23]. Adjuvants may be favoured in peripheral nerve blocks in covid positive patients by increasing the duration and density, thereby decreasing the risk of conversion to GA. The side effects of the adjuvants should be balanced with the benefits. In our survey, the most commonly used adjuvant were opioids, followed by dexamethasone, clonidine, and dexmedetomidine.

Upper limb surgeries require special mention as there are several approaches to brachial plexus blocks (BPB). The specific concerns of BPB include diaphragmatic paralysis, which can worsen the respiratory status in an already compromised patient. This is more common with the inter-scalene and supraclavicular approach and can be reduced by performing phrenic nerve sparing blocks like infraclavicular or axillary blocks [24]. Based on our survey, the most performed approach for upper limb surgery is the supraclavicular approach, probably due to the proceduralist’s experience, as was shown by a series of RA in COVID-19 cases [25].

Only 20% of the anaesthesiologists preferred the use of perineural catheters. This might be because the insertion of perineural catheters is time-consuming and requires frequent patient contact.

COVID-positive patients can have associated hepatic and renal dysfunction, which causes decreased local anaesthetic clearance. This, along with cardiac dysfunction, can theoretically increase the susceptibility to local anaesthetic systemic toxicity (LAST) in these populations. So, measures to reduce and treat LAST, including the availability of intralipid should be ensured in hospitals regularly performing regional anaesthesia. However, 30% of the anaesthesiologists responded that they don’t have intralipid stored in their COVID OT premises.

Viral particles can remain viable in clothes and plastic for a longer duration compared to cardboard. So, it is recommended to use sterile disposable paper drapes when available. In our survey, 75% of anaesthesiologists used disposable plastic sheets, probably because of the lack of paper drapes. In such situations, plastic drapes are preferred over woollen drapes. Antibiotic impregnated drapes though have not proven to have any extra benefits or favourable cost-benefit ratio but have been used by 3% of anaesthesiologists. 

The ultrasound machine surfaces, including the probe, can harbour droplets and can act as a potential source of transmission and should be maintained with strict asepsis in COVID-designated areas. It may be advisable to cover the ultrasound screen and controls with a plastic cover. The probe in use should be ideally covered with a sterile transparent sheath. The various methods being used by the anaesthesiologists who participated in our survey included the use of a camera cover to cover the probes, the use of sterile gloves to cover just the end of the probes, sterile jelly, and chlorhexidine wipes, and povidone-iodine dipping.

The most common method from the survey was cleaning with alcohol-based wipes followed by chlorhexidine wipes. However, alcohol-based disinfection is not recommended for ultrasound transducers. Manufacturer recommendations should be followed for the specific ultrasound machine used. The non-critical areas may be cleaned with gentle soap and water. Transducers should be cleaned with cidex, a glutaraldehyde-based solution, or hydrogen peroxide.

In our survey, the majority of the patients were kept in the OT or shifted directly to the COVID-designated ward at the end of the case, and this is as per the ASRA-ESRA joint statement [4]. 

The ISA position statement [3] mentions the method of sterilization and disinfection of a COVID-designated OT with the use of hydrogen peroxide, 1% sodium hypochlorite, or 75% alcohol wipes for solid surface disinfection. Metallic equipment is to be kept in 1% sodium hypochlorite solution for half an hour, then washed and wiped. However, only half of the anaesthesiologists confirmed proper sterilization methods have been performed in their OT.

The Biomedical Waste Management (BWM) rules in India were amended for COVID-positive patients, and the sharp wastes used for RA, such as needles and syringes, should be collected in puncture-proof tamper-proof containers, autoclaved, shredded and encapsulated, or disposed of [26]. Thirty-nine per cent of the anaesthesiologists responded correctly as recommended by BMW management rules. Autoclaving followed by recycling was another common method being practised as per the survey [27]. 

With many teaching institutes being converted into COVID-designated centres, and the postponement of elective surgeries, the training of residents has been hampered. This is especially a concern in the field of anaesthesia as the majority of the skills are learned by practising in the OT. When asked about the mode of training in RA, the majority of the anaesthesiologists responded that the most common method was web-based learning. This may not be as effective in skill acquisition. Deliberate practice of patients also has been done in some teaching hospitals. However, other effective methods like simulation-based teaching, cadaver/phantom model-based learning, and workshops should be implemented to increase skill acquisition among trainees [28]. 

In our survey, we found out that more than three-fourths of the anaesthesiologists documented anaesthesia management in charts and around 10% in the patient file. Though there are no studies regarding transmission within hospitals via patient files and charts, a better option will be online entries or telecommunication to prevent transmission via fomites.

RA allows for a reduction in airway manipulation, reducing environmental contamination, and reducing opioid and muscle paralysis requirements and should be recommended whenever possible in COVID-19 patients [29].

The limitations of our study are that though it was a pan-India survey, the majority of responses were from Delhi. However, the remaining responses belonged to all the various zones of India, and hence, the data is a reasonable estimate of the prevalent situation in various parts of the country. The strength of our study is its high response rate and that our survey was all-encompassing and is a step in the direction of improving awareness regarding the various aspects of RA practice that are crucial to patient safety during the pandemic.

## Conclusions

In conclusion, the COVID-19 pandemic has led to a substantial increase in preference for RA in comparison to general anaesthesia, especially in COVID-positive or suspected patients. RA practices, sterilization, teaching, and training have also been adapted to the pandemic. However, there is a large scope for improvement in several areas related to RA practice to reduce viral transmission and complications. RA should be used whenever viable, provided it guarantees adequate surgical conditions, ensures patient safety, and provides protection for health workers. The well-established standards specific to RA as well as RA during the pandemic must be adhered to.

## Appendices

Effect of COVID-19 pandemic on regional anaesthesia practices amongst anaesthesiologists of India

Dear all,

Greetings of the day!

Hope we are all keeping safe in this tough COVID -19 pandemic time. We are conducting this survey with the aim to find out the challenges being faced by you regarding regional anaesthesia practice and impact of pandemic on its practice, teaching and training during pandemic time and take your suggestions in improving the same so that the application of regional anaesthesia, teaching and training work is not hampered due to the COVID pandemic.

Please fill the following questionnaire regarding the ‘EFFECT of COVID-19 pandemic on regional anaesthesia practices amongst anaesthesiologists of India’ for your kind views on this important aspect. The data thus generated would help guide future practice and identify any lacunae on this sensitive topic. The form also asks your permission to use related observations as data in this study. The participation is completely voluntary.

Please note that the questionnaire does not ask for your identity, so anonymity will be ensured and no personal or institutional details would be disclosed.

Thanking you in anticipation. Best Wishes!

*Required

1.    Email *

2.  Consent to participate: I have reviewed the information provided in the    * participation information sheet provided above and have made required clarification if required from the investigator. I understand that my participation

in this survey is voluntary , and I can decline my participation without giving any reason. By clicking on the "I Agree button", I give consent to be part of the study.

Mark only one oval.

I agree

General information

3.      Type of institution you are affiliated to * (Mark only one oval.)

Medical college (Private/government/autonomous) Government non-teaching hospital

Corporate Hospital

Free Lancing Practitioner

Others

4.   State of India that you work in *

5.   Years of Experience (Mark only one oval.)

Less than 5 years 5-10 Years

10-15 Years

More than 15 Years

6.  What Percentage of Surgeries are done under Regional Anesthesia Techniques   * in your Department? (Mark only one oval.)

Less than 20%

20-40%

40-60%

More than 60%

7.  What percentage of patients undergoing surgery receive regional anesthesia       * techniques for postoperative analgesia? (mark only one oval.)

Less than 10%

10-20%

20-30%

More than 30%

8.  Is there a change in the incidence of using regional anesthesia techniques for     * surgery in your department during COVID-19 Pandemic ?

Increased/  Decreased \No change

9. Has there been a change in an overall practice of regional anaesthesia with * regards to the utilization, use of PPE, asepsis, disinfection, monitoring, type of technique used for regional anaesthesia techniques for postoperative analgesia in your institute

Yes, there is some change

No, there is no change

10.  What mode of anesthetic technique do your department prefer in COVID 19       * diagnosed / suspected patients undergoing surgery?

General anaesthesia /Regional anesthesia/No preference for any technique over the other

11.   If there is a preference, is there a difference in asking COVID-19 testing in         * cases scheduled under regional versus general anaesthesia?

Testing is more stringent for GA cases/ No difference

12.  Is a designated Operation theatre available to perform surgeries of COVID         * confirmed/suspected patients in your institution?

(Mark onlyI one oval.)

Yes , with negative Pressure system

Yes, but with no negative Pressure system No

13. Which of the following Preoperative investigations are routinely done in all * COVID-19 confirmed/suspected young adult (18-60) ASA-I patients posted for surgery under regional anaesthesia? (Tick all that apply)

Total Leucocyte count with Hb Platelet count

Coagulation profile Inflammatory markers Chest Xray

CT chest ECG

Renal function tests Liver function tests 2D Echo

Room air saturation Temperature Other:

14.  Do you routinely provide sedation for all COVID 19 confirmed/ suspected  * patients undergoing surgery under regional anesthesia?

Yes, in all cases

Avoided unless required

15.               How do you supplement Oxygen for COVID 19 confirmed/ suspected patients   * undergoing surgery under regional anesthesia?

Mark only one oval.

Oxygen supplementation avoided

Using a Nasal Canulla/facemask with a flow rate upto 2L/min

Facemask with a flowrate of 2-4L/min Facemask with a flowrate more than 4L/min Other

16. Is a surgical/three ply/N95 mask applied to COVID 19 confirmed/ suspected     * patients undergoing surgery under regional anesthesia

Yes, a surgical mask

Yes, an N-95 mask/FFP3 mask No

17.    What type of personal protective equipments are used by the operators * performing central neuraxial block on COVID 19 patient? (can choose more than one option)

Tick all that apply.

Level 3 PPE N95 MASK

face shield Eye goggles

Impermeable Gown Double gloves Shoe cover

Other:

18. How do you document the anesthesia management in a Covid-positive patient in your hospital? (Mark only one oval.)

Anesthesia chart Patient file Online entry

Documentation is done outside in the non-covid area over telephonic conversation

Central Neuraxial blockade and peripheral blockade

19.     What position do you perform neuraxial techniques in a COVID-19 patient? (Mark only one oval.)

Lateral Sitting

position depends on patient

20.  What needle size is used to perform spinal anesthesia in COVID-1 9 PATIENT? (Mark only one oval.)

23G

25G

26G

27G

21.  What needle size is used to perform epidural anesthesia in COVID19 PATIENT? (Mark only one oval)

16G

18G

Whichever is available

22. What type of needle do you use for spinal anesthesia in a COVID-1 9 patient? * (Mark only one oval)

Quinckie tip needle Pencil tip needle

23. How do you disinfect the skin prior to performing neuraxial blocks in COVID-19 * patients? (Mark only one oval.

Providone iodine

Chlorhexidine with Isopropyl Alcohol Sterillium - Alcohol based disinfectant Both Povidone iodine and Chlorhexidine

24.  What kind of drapes are used in your institution for COVID-19 patients for * perfoming regional anesthesia? (Mark only one oval.)

Disposable plastic drapes/ Disposable paper drapes/ Reusable woolen drapes/ Antimicrobial impregnated drapes

25.   Who generally perfoms the block in COVID-1 9 positive patients? (Mark only one oval.)

Consultant/ Senior Resident/Post graduate Trainee

26.     What method do you use for epidural space identification in COVID 19              * Patients? (Mark only one oval)

Loss of resistance to Air/ Loss of resistance to Saline/ Hanging drop method/ Ultrasound/Others

27. Which adjuvants do you use in spinal anesthesia for COVID-19 patients? (can choose more than 1, Tick all that apply.)

Morphine Fentanyl Clonidine

Dexmeditomedine

No adjuvants are used

Other adjuvants or combination

Other:

28.               What prophylactic measures do you use to prevent hypotension following         * spinal anesthesia in a COVID-19 patient?

No active interventions

Prophylactic vasopressors

Preloading

Coloadin

29.               What are the Issues you have faced while performing a Central neuraxial or       * peripheral nerve block with PPE? Can choose more than one. (Tick all that apply)

Poor vision due to fogging

Difficulty in palpation and identifying the intervertebral spaces Multiple needling attempts

Increased duration of performance Failure of block

Increased incidence of dural puncture while performing epidural

30.   Which of the following methods would you use to treat PDPH in COVID positive * patients refractory to conservative management in your hospital? (Tick all that apply.)

Sphenopalatine ganglion block/  Epidural blood patch/Bilateral Greater occipital nerve block/ Others

31. How do you use epidural analgesia for postoperative pain relief in COVID  * positive patients in your hospital? (Mark only one oval.)

Yes, Repeated bolus at regular intervals/ Yes, as Continous infusion/Bolus when patients complaints/ Patient controlled Analgesia

32. Which investigations will you check prior to removal of epidural catheter? * (Tick all that apply)

Platelet count /Coagulation profile/ Others

33.               is General anesthesia provided prior to performing regional blocks in COVID      * patients? (Mark only one oval.)

Yes/ No

34.       Which technique do you commonly use to perform peripheral nerve blocks in    * COVID positive patients? (Mark only one oval.0

USG guided landmark

Nerve stimulator

Combined USG with nerve stimulator

35.                Which adjuvant do you commonly use for performing peripheral nerve blocks    * in COVID positive patients? (Tick all that apply.)

opiods Clonidine

Dexmedetomidine

Dexamethasone

Other:

36.              How do you disinfect the skin prior to performing peripheral nerve blocks in     * COVID positive patients? (Mark only one oval)

Povidone iodine

Chlorhexidine with isopropyl alcohol

Pure alcohol based solutions

Other:

37.   Is intralipid stored inside the COVID designated OR in your hospital? * (Mark only one oval)

Yes/No

38.              What issues have you had faced in performing peripheral nerve blocks with PPE? Can choose multiple options: (Tick all that apply)

Poor visibilty due to fogging Poor ultrasound images wrong side block

increased performnace time increased failure rates

Increased volume of local anesthetic use IV injection

multiple attempts Nerve injury Hematoma Other:

39.             Do you insert perineural catheters for postop analgesia in COVID positive         * patients in your hospital

No/Yes

40. If using, what is the Duration for which you keep perineural catheters in COVID positive patients?

Less than 24 hours

24-72hours

More than 72 hours

41.              For upper limb surgeries which among the following blocks is commonly          * preferred in your hospital for COVID positive patients?

Interscalene nerve block /Supraclavicular nerve block/ Infraclavicular nerve block/ Axillary nerve block /Individual nerves are blocked

42.              Measures to maintain ultrasound sterilty in COVID designated OR in your hospital (Tick all that apply)

Camera cover

Antibiotic impregnated plastic Cover

Sterile gloves Sterile jelly Chlorhexidine rubs

Dipping into Povidone iodine

None Other:

43.   How do you disinfect USG after use in a COVID positive patient? (Mark only one oval)

Alcohol based Wipes Chlorhexidine based Wipes Glutaraldehyde ( CIDEX) Hydrogen peroxide

Ultraviolet light based sterilisation

Soap and water

None

Other:

Postoperative period and recovery

44. Where do you monitor COVID Positive patients who underwent surgery under    * regional anesthesia in the immediate postoperative period? (Mark only one oval.)

Within the COVID designated OR PACU

COVID designated ward

45.  How is the Sharps like needles and syringes used in covid positive patients disposed in your hospital? (Mark only one oval)

Incineration/ Collected in separate tamper proof bags/autoclaved and recycled /Collected in separate tamper proof bags/ autoclaved and shredded /Either recycled or shredded/Other

46. What are the methods for cleaning the operating room after the surgery of a    * COVID positive patient in your hospital? (Tick all that apply)

Fogging with Hydrogen peroxide vapour followed by segregation wiping floors and solid surfaces with sodium hypochlorite solution wiping floors and solid surfaces with 75% alcohol/ Disinfection of metallic equipments with sodium hypochlorite solution/Other:          

47.  What are the training methods provided for residents for performing regional    * anesthesia in your hospital during the peak of COVID pandemic when elective surgeries are stopped?(Tick all that apply)

Simulation based

Deliberate practice on patients under the supervision of faculty Web based learning

Cadaver/phantom model based learning Workshops Other:

48.  Thank you for your response. Additional remarks if any.
